# From Insect Pheromones to Mating Disruption: Theory and Practice

**DOI:** 10.3390/insects12080698

**Published:** 2021-08-03

**Authors:** Giovanni Benelli, Andrea Lucchi

**Affiliations:** Department of Agriculture, Food and Environment, University of Pisa, Via del Borghetto 80, 56124 Pisa, Italy; giovanni.benelli@unipi.it

## 1. Introduction

Insects perceive and integrate a hierarchy of visual, chemical and tactile cues for feeding and reproductive purposes, as well as for predator and parasitoid avoidance. Among semiochemicals routing insect’s decisions, pheromones play a key role in mediating intraspecific communication [1,2]. The study of insect chemical ecology with special reference to their pheromones is a fascinating research field. Pheromone-mediated mating disruption represents an effective and eco-friendly biocontrol technique to manage insect pests of agricultural importance. Worldwide, agricultural pests affecting more than 800,000 hectares of crops are managed by mating disruption [3]. This technique relies on the release of synthetic sex pheromones from dispensers in crops, interfering with mate finding and reproduction of the pest through both competitive and non-competitive mechanisms [4,5]. Unfortunately, the use of mating disruption is still restricted to a rather limited number of crop pests, with special efforts being directed toward moths [3]. However, the mating disruption potential is huge and urgently needs to be explored further.

## 2. Insect Chemical Ecology: From the Laboratory to the Field

In this framework, the Special Issue “From Insect Pheromones to Mating Disruption: Theory and Practice” includes both laboratory and field studies on insect pheromones, as well as on mating disruption efficacy against insect pests of economic importance.

Herein, the following topics have been covered:

**(a)**  **Pheromone biology**, with special reference to the various factors, often overlooked, affecting pheromone production in species of economic importance, including wood boring beetles, e.g., *Lyctus africanus* Lesne (Coleoptera: Bostrichidae) [6]; a further focus has been provided on the citrophilous mealybug, *Pseudococcus calceolariae* (Maskell) (Hemiptera, Pseudococcidae), assessing the potential negative impact of male multiple matings on mass trapping and mating disruption [7].
**(b)**  **Development of novel mating disruption tools and approaches**. Several novel mating disruption approaches have been developed and/or optimized to manage a broadly diverse number of insect pests. A major focus has been devoted to moth pests, showing carefully conducted mating disruption experiments on grape (*Cryptoblabes gnidiella* (Millière, 1867) (Lepidoptera: Pyralidae)) [8], plum (*Grapholita funebrana* Treitschke (Lepidoptera: Tortricidae)) [9], almond (*Amyelois transitella* (Walker) (Lepidoptera: Pyralidae)) [10], and tomato (*Helicoverpa armigera* (Hubner) (Lepidoptera: Noctuidae)) [11], as well as on polyphagous leafrollers, such as *Proeulia auraria* (Clarke) (Lepidoptera: Tortricidae) [12]. Concerning mealybugs, further research efforts have been directed to evaluating the potential of *P*. *calceolariae* mating disruption in apple and tangerine orchards [13].
**(c)**  **Reviews on insect chemical ecology**. The Special Issue ends with two broad reviews. The first summarizes current knowledge on insect sex pheromone research and its application in Integrated Pest Management [14]. The second review offers an updated analysis of what we really know on tephritid fruit fly semiochemicals, their real-world applications and the related research challenges [15].

## 3. The Future

Overall, we enjoyed organizing the Special Issue “From Insect Pheromones to Mating Disruption: Theory and Practice” greatly, and sincerely thank all the authors for their fine contributions. On the other hand, we are very aware that the present Special Issue cannot reflect the wide diversity of topics and challenges currently characterizing the pheromone and MD research. In particular, further research efforts are still needed to develop novel approaches to combat emerging pests, with special reference to invasive species, to fully clarify the mechanisms of action of MD-based control tools, to shed light on potential non-target effects, as well as to boost the efficacy of MD programs through the optimization of release geometries.
